# Techniques to estimate colostrum quality and the effects of cow age and prepartum supplement intake levels on colostrum quality and serum IgG levels

**DOI:** 10.1093/tas/txaa121

**Published:** 2020-12-22

**Authors:** Julia M Dafoe, Samuel A Wyffels, Cory T Parsons, Boone H Carter, Timothy DelCurto, Darrin L Boss

**Affiliations:** 1 Northern Agricultural Research Center, Montana State University, Havre, MT; 2 PerforMix Nutrition Systems, Nampa, ID; 3 Department of Animal and Range Sciences, Montana State University, Bozeman, MT

## INTRODUCTION

In cattle, ingestion of sufficient quality and quantity of colostrum following birth is essential for the future health and performance of the newly born calf (Rauprich et al., 2000). Colostrum quality, as measured by its immunoglobulin (IgG) content, can vary among cattle within a herd and across breeds (Gulliksen et al., 2008). Factors that are often cited as impacting passive transfer in calves are timing of colostrum ingestion, method of administration and volume of colostrum, IgG concentration, age of the dam, and plane of nutrition including mineral and supplementation intake of the dam (Weaver et al., 2000). The ingestion and absorption of adequate IgG are called passive transfer and allow the newborn calf to build natural immunity for improved lifetime health and production (Calloway et al., 2002), and failure of passive transfer is a condition that predisposes the newborn calf to the development of diseases (Weaver et al., 2000).

Numerous tools are available to measure on farm colostrum quality; these include hydrometer/colostometer, digital refractometer, and optical refractometers. Total serum protein, a blood serum test to assess the absorption of adequate quality and quantity of colostrum, can also be assessed using both the digital and optical refractometer in newborn calves (Penn State University, 2016). A colostrum % Brix value of >22 is considered to contain adequate IgG to support effective passive transfer insuring the future health and production of the calf (Elsohaby et al., 2015).

The objectives of this study were to 1) determine the impacts of differential protein supplement intake during gestation with winter grazing beef cattle on postcalving colostrum quality and total serum protein of both cows and calves and 2) evaluate the effectiveness of a digital vs. optical Brix refractometer at measuring the colostrum quality of beef cattle. Our hypotheses were: 1) differential protein supplement intake would have no impacts on colostrum quality or total serum protein in multiparous beef cattle and 2) optical Brix refractometers are an economical alternative to Digital Brix methods to assess colostrum quality.

## MATERIALS AND METHODS

The use of animals in this study was approved by the Institutional Animal Care and Use Committee of Montana State University AACUC #2018-AA12.

Research was conducted at Montana State University’s Northern Agricultural Research Center, Havre, MT. Multiparous Artificial Insemination (AI) bred (June 2, 2018) Angus cows (*n* = 45) 4 to 9 yr of age at Montana State University’s Northern Agricultural Research Center near Havre, MT had free-choice access to a 28.7% Crude Protein self-fed pressed block supplement with a target daily intake range of 0.45 to 0.91 kg/cow/d. The supplement included salt (23%), bitterness, and hardness as mechanisms to limit supplement intake (Table 1). Cows had ad libitum access to the supplement starting in their second trimester (129 d of pregnancy) through parturition; however, intakes were only measured from d 164 to 215 of pregnancy. Intakes were measured using eight feeding stations in a SmartFeed Pro self-feeder system (C-Lock Inc., Rapid City, SD).

**Table 1.** Bovibox HM supplement composition for cattle winter grazing rangeland in 2018 to 2019 at the Thackeray Ranch, Havre MT (as-fed basis)

Crude protein	28.7% min
Crude fat	1.45% min
Crude fiber	5.0% max
Calcium	1.3% min
	1.8% max
Phosphorus	0.7% min
Salt	23% min
	26% max
Potassium	1.5% min
Magnesium	2.5% min
Manganese	856 ppm min
Zinc	1,074 ppm min
Copper	213 ppm min
Copper (from chelate)	108 ppm min
Cobalt	15 ppm min
Iodine	26 ppm min
Selenium	3.3 ppm min
	3.6 ppm max
Selenium yeast	—
Vitamin A	12,000 IU/lb
Vitamin D	4,000 IU/lb
Vitamin E	25 IU/lb
NPN not more than	9.7%

NPN = Non-protein nitrogen.

Cows were categorized as either low (>−0.75 SD from mean), average (±0.50 SD from mean), or high (>+0.75 SD from the mean) supplement consumers based on calculated supplement intake during d 164 to 215of pregnancy. Only cows that conceived first service AI pregnancy were used to ensure data collection occurred within similar time during the calving season. Twenty cows of each intake group were randomly identified and observed for signs of calving and the first 15 cows in each group to calve were selected for the study. Average daily supplement intakes were 0.12, 0.32, and 0.67 kg/cow/d, respectively, and both the low and average intake treatment were below the target label daily-recommended intake range of 0.45 to 0.91 kg/cow/d.

All cows in this study were housed together in a small pasture and observed hourly for signs of parturition. If no assistance was needed the pair was left in the pasture until data were collected from the dam. Once parturition occurred, calves were observed to ensure they had nursed within 2 h of birth. Cows that had calved within the last 12 h were brought into the barn at 7 a.m. or 7 p.m. A 50 mL colostrum sample was collected uniformly from all 4 teats, composited and sampled. Colostrum quality was measured using an optical Brix scale (Bellingham + Stanley, Lawrenceville, GA 30043) and a digital Brix scale (Misco Palm Abbe, Solon, OH) using methods outlined by Quigley et al. (2013). Due to the extreme cold weather, calves in each treatment group (*n* = 3 for high, *n* = 2 for average, and *n* = 4 for low) were brought into the barn and treated for hypothermia (rectal temperature of less than 36 °C). Hypothermic calves were treated with 50% Dextrose solution, placed in warm environment, and fed colostrum from their dam to ensure calves received adequate colostrum within 2 h of birth.

At time of milk collection, a 10 mL blood sample was collected from the dam via venipuncture utilizing vacutainer tube without anticoagulant. Serum was separated by centrifugation within an hour of collection; the sample was spun at 2100 rpm for 15 min. The serum sample was tested for total protein (Tpr g/dL) using digital refractometer. Calf blood samples (10 mL) were collected 3 d postpartum via venipuncture utilizing vacutainer tube without anticoagulant. Serum was separated by centrifugation within an hour of collection; the sample was spun at 2100 rpm for 15 min. The serum sample was tested for total protein (Tpr, g/dL) using digital refractometer.

Data were analyzed using generalized linear models in an analysis of variance evaluating the effects of supplement intake levels and cow age on % Brix of colostrum, and Tpr content of cow and calf serum. Linear regression was used to evaluate the relationship of the two refractometer methods, the relationship of colostrum level to cow serum, and calf serum 3 d postpartum, and % Brix of colostrum to hours postpartum that colostrum was collected. Least square means were separated using the Tukey method when *P* < 0.05. Cow was considered the experimental unit and all statistical analyses were performed in R (R Core Team, 2017).

## RESULTS

**Figure 1.**
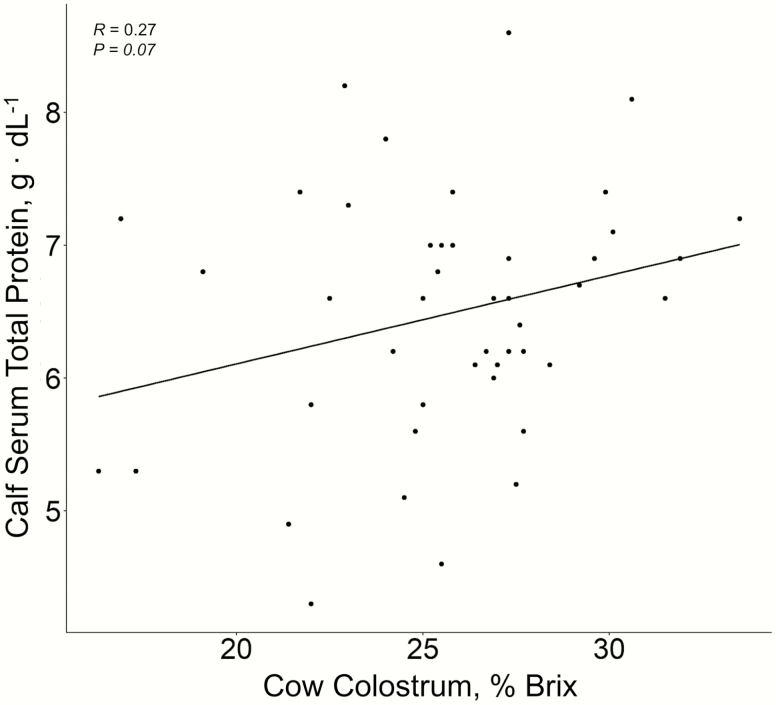
Relationship of cow colostrum obtained within 12 h postpartum to calf blood serum taken 3 d postpartum with multiparous spring calving commercial Angus beef cattle.

Beef cattle age and supplement intake level did not interact with colostrum % Brix, nor cow and calf serum Tpr (*P* ≥ 0.29). As a result, data are presented as main effects relative to supplement intake level (Table 2) or cow age (Table 3). There were no differences between supplement intake levels on estimated colostrum quality as measured using a digital refractometer (*P* = 0.35; Table 2). In addition, cow age and supplement intake had no effect on Tpr of cow serum taken within 12 h of parturition nor Tpr of calf serum taken at d 3 postpartum (*P* ≥ 0.14). However, Tpr of calf serum tended (*P* = 0.07) to be related to colostrum % Brix levels although the relationship was minimal (*R* = 0.27; Fig. 1). No relationships (*P* = 0.73) were observed between colostrum % Brix and Tpr of cow serum at parturition.

**Table 2.** Influence of protein supplement intake during gestation on beef cattle weight and body condition as well as postpartum colostrum quality, cow blood serum, and calf blood serum

	Protein supplement intake^*a*^			SE	*P*-value
	Low	Average	High		
Cow weight, kg					
Initial, d 129 gestation	607.4	594.1	588.3	13.95	
d 215 gestation	630.3	622.9	627.8	12.40	
Cow BCS, 1–9 scale					
Initial, d 129 gestation	5.67	5.65	5.42	0.09	
d 215 gestation	5.67	5.58	5.27	0.09	
Colostrum, (%) Brix^*b*^	26.2	24.7	26.7	0.99	0.34
Cow serum (Tpr)^*b*^	6.92	6.51	6.98	0.14	0.22
Calf serum (Tpr)^*c*^	6.16	6.59	6.67	0.23	0.30

BCS = Body Condition Score.

^*a*^Cows were categorized as either low (>−0.75 SD), average (±0.50 SD), or high (> +0.75 SD) based on mean daily supplement intake.

^*b*^Cow serum and colostrum were collected within 12 h of parturition (average collection time was 5.25 h after parturition).

^*c*^Calf serum was collected 3 d after birth.

**Table 3.** Effect of cow age on body weight, body condition, colostrum quality, cow serum, and calf serum

	Cow age^*a*^		SE	*P*-value
	Young^*a*^	Old^*b*^		
Cow weight, kg				
Initial, d 129 gestation	568.3	623.6	9.97	
d 215 gestation	604.0	649.3	9.02	
Cow BCS, 1–9 scale				
Initial, d 129 gestation	5.42	5.73	0.09	
d 215 gestation	5.53	5.58	0.08	
Colostrum, % Brix^*b*^	25.1	26.3	0.80	0.94
Cow Serum (Tpr)^*b*^	6.67	6.94	0.11	0.62
Calf Serum (Tpr)^*c*^	6.15	6.80	0.19	0.14

^*a*^Young cows = 4, 5, and 6 yr; old cows = 7 and 8 yr.

^*b*^Cow serum and colostrum were collected within 12 h of parturition average collection time was 5.25 h after parturition.

^*c*^Calf serum was collected 3 d postpartum.

Cows were divided into two age classes, young (4 to 6 yr) and old (7 and 8 yr) to analyze impacts of age on colostrum % Brix content, and serum (Tpr) of both cow and her calf (Table 3). There were no differences between age class of cow on colostrum % Brix (*P* = 0.94), cow serum Tpr (*P* = 0.62), and calf serum Tpr (*P* = 0.14).

**Figure 2.**
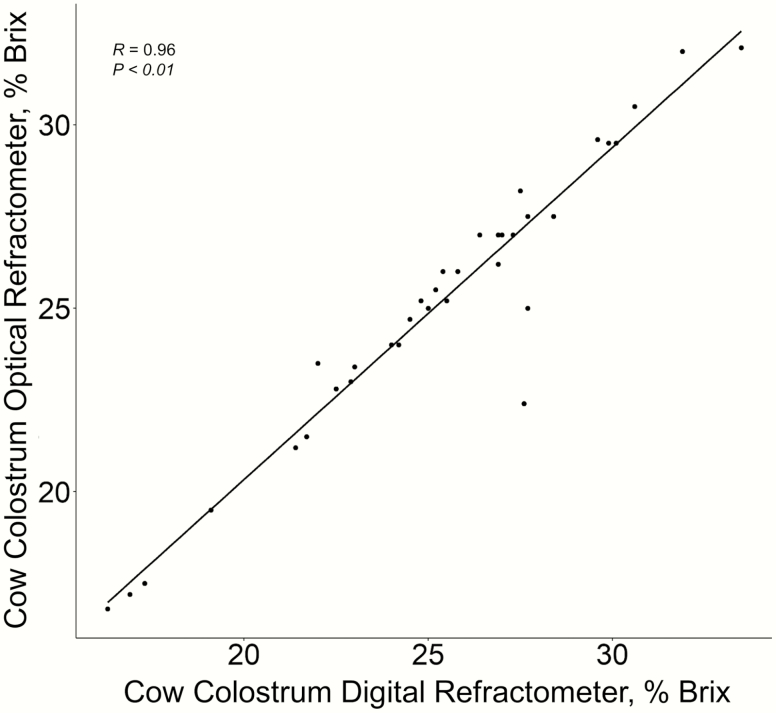
Comparison of digital vs. optical refractometers to assess colostrum quality in beef cattle with samples taken within 12-h postpartum.

The optical refractometer was found to be a good alternative to the more expensive digital refractometer. When regressing values of the two techniques, they were observed to be similar (*P* < 0.01) and both gave values that were closely related (*R* = 0.96; Fig. 2).

## DISCUSSION

**Figure 3.**
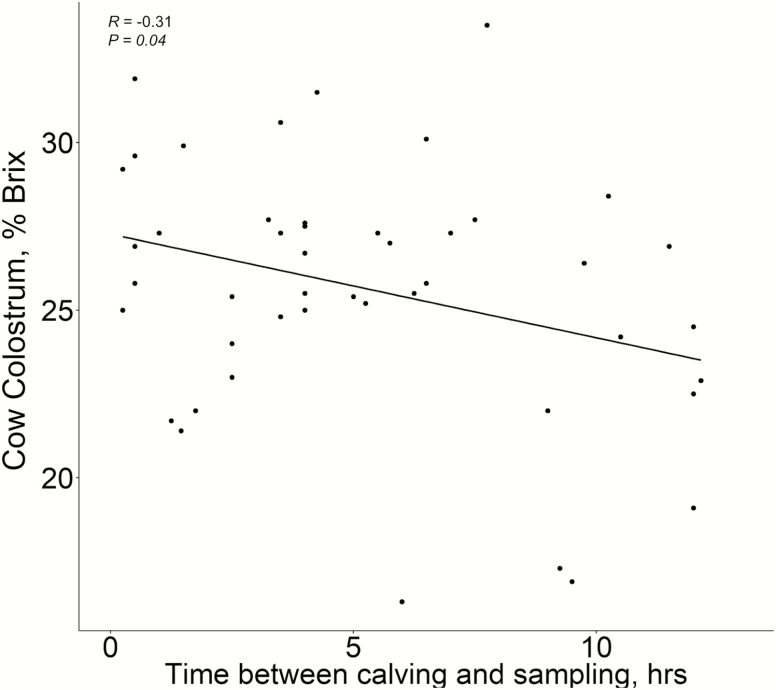
Correlation between colostrum Brix score and time of birth with multiparous, spring calving Angus cows.

A colostrum % Brix value of >22 is considered to contain adequate IgG to support effective passive transfer and future health and production (Elsohaby et al., 2015). Therefore, the values observed in this study suggest that most cows met or exceeded the IgG levels needed to insure proper passive transfer for calf health, provided the calves consumed the colostrum in a timely manner postpartum (Fig. 1); Buczinski and Vandeweerd, 2016). We did not observe any influence of gestational supplement intake level or cow age on colostrum quality estimated with digital refractometers. This may be due to the relatively good body condition of the cows during the winter grazing part of gestation demonstrated by body condition scores greater than 5 at the end of the study period (Tables 2 and 3) and at calving. In addition, from d 215 of gestation till calving, cows were fed a moderate quality total mixed ration and, when combined with the protein supplement, likely met or exceeded their nutritional needs. Simply put, the relative condition of the cows at birth likely limited our ability to see differences in colostrum quality due to differences in supplement intake. In addition, the colostrum Brix score decreased as time from birth increased (*R* = −0.31, *P* = 0.04; Fig. 3) as also reported by Foley and Otterby (1978) where colostrum IgG decreased slightly in the first 24 h and sharply afterwards in dairy cows. The decline in Brix values as a function of hour postpartum were small as well as highly variable and, as a result, did not necessarily contribute to the lack of statistical inference with colostrum quality.

## IMPLICATIONS

Cow age and supplement intake levels of mature cows during the mid to late stages of gestation did not influence colostrum quality at birth. The lack of colostrum quality differences was likely due to the moderate to good condition of the cows and adequate nutrition 90 d prior to parturition. Optical refractometers are an economical alternative to digital refractometers.



*Conflict of interest statement*. None declared.
